# FcLRR1 Regulates Hyphal Growth and Plant Infection in *Fusarium circinatum*

**DOI:** 10.3390/jof12040282

**Published:** 2026-04-16

**Authors:** Tingting Dai, Chao Chen, Fangyi Ju, Jiahui Zang, Zhongqiang Qi, Haiwen Wang, Xiaorui Zhang, Chun Yang

**Affiliations:** 1 Co-Innovation Center for Sustainable Forestry in Southern China, Nanjing Forestry University, Nanjing 210018, China; chaochen0520@163.com (C.C.); 15850535371@163.com (F.J.); zjh20011009@163.com (J.Z.); 13865744264@163.com (H.W.); 17761709897@163.com (X.Z.); yangchun0724@163.com (C.Y.); 2 Institute of Plant Protection, Jiangsu Academy of Agricultural Science, Nanjing 221000, China; qizhongqiang2006@126.com

**Keywords:** pitch canker disease, *Fusarium circinatum*, *FcLRR1*, pathogenicity

## Abstract

Pitch canker caused by the fungus *Fusarium circinatum* is a destructive disease that affects pines in Europe, South Africa, and North America, particularly along the southeastern and western coasts of the United States. This study systematically elucidated the function of the Leucine-rich repeat (LRR) protein FcLRR1 in the pine pitch canker pathogen *Fusarium circinatum*. A total of 13 LRR proteins were identified via bioinformatic analysis. Using a gene knockout system, we demonstrated that deletion of FcLRR1 significantly impaired vegetative growth, conidiation, and conidium germination; led to a complete loss of macroconidia production; and drastically reduced abiotic stress tolerance and virulence. Transcriptome profiling revealed 612 downregulated genes, which were significantly enriched in pathways such as starch and sucrose metabolism, indicating that *FcLRR1* modulated energy supply and pathogenicity through carbon source utilization. Through genome-wide protein structure modeling and yeast two-hybrid assays, we identified and validated the interaction between FcLRR1 and ALG-11, among other candidate proteins, further supporting its involvement in carbon metabolism, cell wall integrity, and pathogenesis. This study represents the first functional characterization of an LRR-containing protein in a forest pathogenic fungus and provides a foundational basis for developing targeted disease control strategies.

## 1. Introduction

*Fusarium circinatum*, a filamentous fungal pathogen, is the causative agent of pine pitch canker (PPC), which poses a significant threat to pine (*Pinus* spp.) plantations worldwide. This pathogen infects a wide range of pine species, resulting in substantial ecological and economic damage. *F. circinatum* Nirenberg & O’Donnell was first identified on *Pinus virginiana* in the United States [1]. Since then, it has spread to several other regions, including Africa, Asia, South America, and southern Europe [2]. Its potential for severe outbreaks, especially in forest nurseries and plantations, has made it a focus of quarantine regulations in various areas [3]. *F. circinatum* was initially classified within the *F. subglutinans* species complex, and advances in molecular biology, including multilocus sequence typing (MLST), have confirmed that *F. circinatum* is a distinct species [4].

*F. circinatum* primarily infects several species of pine, with *Pinus radiata* being the most susceptible species (potential interactions). It also infects *P. elliottii*, *P. patula*, and more than 80 other species of *Pinus* [5], causing extensive damage to forests and nurseries. Furthermore, artificial inoculation studies have demonstrated that *F. circinatum* can infect various plant genera, including Abies, Larix, Libocedrus, and Picea [6,7,8]. This pathogen impacts pine development at all stages. As a seed-borne pathogen [9], it can lead to mortality in seeds and seedlings, causing both pre- and postemergence damping-off, as well as decay in lignified seedlings (late damping-off) [10]. Infected trees exhibit needle discoloration, defoliation, and the formation of resinous cankers on trunks and branches, which can ultimately result in the death of mature trees. Seedlings are particularly vulnerable and often succumb to disease before reaching maturity. In regions such as Spain [11] and South Africa [12], *F. circinatum* poses a significant threat to commercial forestry, leading to substantial economic losses.

Leucine-rich repeat (LRR) proteins represent a large and diverse family characterized by their repetitive LRR motifs, which typically consist of 20 to 30 amino acids, featuring a conserved leucine residue at every second position [13,14]. Initially, Kobe et al. classified these proteins into seven subfamilies: the ribonuclease inhibitor-like family, SDS22-like family, cysteine-containing family, bacterial family, typical family, plant-specific family, and *Treponema pallidum* family [15]. Recent studies have identified the eighth category—TpLRR/BspA-like LRRs—distinguished by β-strands located on the convex surface of the solenoid structure, the absence of helical motifs on the concave surface, and a unique sequence conservation pattern (e.g., C/NxxLxxIxLxxxLxxIgxxAFxx) [16]. LRR domain proteins have been identified in nearly all organisms and play critical roles in processes related to innate immunity, ubiquitin-mediated cellular functions, apoptosis, autophagy, nuclear mRNA transport, neuronal development, and the pathogenesis of various pathogens [15,17,18].

To defend against pathogen invasion, both plants and animals have evolved nucleotide-binding domain leucine-rich repeat receptors (NLRs) that detect pathogens and initiate immune responses [19,20]. In plants, LRR proteins play crucial roles in recognizing pathogen-associated molecular patterns (PAMPs) [21] and activating defense responses. For example, LRR receptor-like kinases (LRR-RLKs) are essential for perceiving pathogen signals and initiating defense mechanisms [22]. Additionally, LRR-containing proteins are essential components of immune receptor complexes that mediate resistance against a wide variety of pathogens, including bacteria and fungi [23,24]. Recent studies have highlighted the role of LRR proteins in regulating root development, hormone signaling, and responses to abiotic stress [25,26,27]. In animals, the LRR-containing inflammasome NLRP3 plays a critical role in various diseases affecting the human body because of its capacity to be activated by a diverse array of pathogens and danger signals [28,29]. These diseases include familial periodic autoinflammatory syndromes, type 2 diabetes, Alzheimer’s disease, asthma, allergic airway inflammation, myocardial infarction, and atherosclerosis [30].

Compared with their well-characterized counterparts in plants and animals, LRR-containing proteins in fungi remain underexplored. Comparative genomic analyses revealed that fungal genomes encoded fewer LRR-containing proteins, most of which lacked the canonical pattern recognition receptor (PRR) architectures typically associated with immune surveillance in other eukaryotes. This distinction may reflect the unique evolutionary trajectory of fungal immune recognition mechanisms [31]. Nevertheless, fungi possess specific LRR-containing proteins that play crucial roles in signal transduction pathways. For example, in *Saccharomyces cerevisiae* [32], the LRR domain of adenylate cyclase functions as a sensor for glucose availability, modulating cyclic adenosine monophosphate (cAMP) levels to regulate metabolic adaptation and stress responses. Similarly, *Candida albicans* utilizes the LRR-containing adenylate cyclase Cyr1 to detect peptidoglycan fragments derived from serum, which triggers cAMP-dependent morphological transitions from yeast to hyphal growth, a phenotypic switch associated with virulence [33]. These examples highlight the diverse functional repertoire of fungal LRR proteins, which diverge from classical PRR-mediated immunity while still playing crucial roles in environmental sensing and adaptation to pathogens.

Although the whole-genome sequence of *F. circinatum* has been established, the molecular processes underlying its pathogenic behavior remain poorly characterized [34]. While numerous studies have identified candidate genes and gene clusters that may be involved in growth and pathogenicity [35,36], only RAS2, FUB1, and *Fcrho1* have been experimentally validated to influence fungal development and virulence [37,38,39]. Furthermore, the functional roles of LRR proteins in the biology of *F. circinatum* remain entirely unexplored. To address this knowledge gap, this study aimed to characterize the role of the LRR protein FcLRR1 in *F. circinatum*. Using a highly efficient and stable genetic transformation system developed in our previous research, we constructed *FcLRR1* knockout and complemented mutants through homologous recombination. The FcLRR1 deletion mutants and the wild-type strains were subjected to comprehensive phenotypic assays, including assessments of fungal growth, sporulation, conidial germination, and pathogenicity. The findings from this investigation will enhance our understanding of the molecular mechanisms governing growth and virulence in *F. circinatum* and potentially other fungi within the same genus.

## 2. Materials and Methods

### 2.1. Fungal Material and Growth Conditions

The *F. circinatum* strain A015-1 isolated from Nanjing Customs (Nanjing, China) was used for genetic transformation and knockout in this study. The two additional strains used in this study represented LRR knockout (∆*FcLRR1*) and complement (*FcLRR1-c*) mutants of A015-1, both of which were generated during the course of this research (see below). Which were deposited in the Forest Pathology Laboratory of Nanjing Forestry University. All the strains were routinely cultured on potato dextrose agar (PDA) media at 25 °C in the dark.

### 2.2. DNA and mRNA Extraction and cDNA Preparation

The genomic DNA was extracted from strains cultivated on PDA media plates for five days via the cetyltrimethyl ammonium bromide (CTAB) method [40]. The DNA extract concentration was determined via a microspectrophotometer (BioPhotometer^®^, Eppendorf, Hamburg, Germany), with the A260/A280 ratio maintained within the optimal range of 1.8–2.0 to ensure purity.

The mycelia of 5-day-old vegetative fungus strains or plant samples were ground in liquid nitrogen for each strain separately in a sterile mortar, and the total RNA of strain A015-1 and all the mutants was extracted via an RNA extraction kit (Biotech, Shanghai, China). Then, the genomic DNA was removed with DNase, an RNase inhibitor (TaKaRa, Dalian, China) and other reagents. The integrity, purity, and quality of the total RNA were assessed via agarose gel electrophoresis and a Nanodrop microspectrophotometer. The RNA integrity was considered high if the 28S:18S rRNA ratio was approximately 2:1. For RNA purity, an optimal A260/A280 ratio was maintained between 1.8 and 2.0. The remaining RNA was stored at −70 °C.

A Prime ScriptTM Double Strand cDNA Synthesis Kit (TaKaRa, Dalian, China) was used to reversely transcribe the mRNA into the cDNA of *F. circinatum.* The cDNA was stored at −20 °C for long-term use.

### 2.3. Identification and Analysis of LRRs Genes

Using genomic and transcriptomic data from *F. circinatum* (pine resin canker pathogen), this study systematically identified leucine-rich repeat (LRR) domains through bioinformatics approaches, including BLAST 2.2.31+ and HMMER 3.3.2.

The protein domains were predicted and analyzed via the SMART (accessed on 17 March 2026) online tool (https://smart.embl.de/), which focused specifically on the number, distribution, and tandem arrangement of leucine-rich repeat (LRR) domains within the *Fcgrr1* gene. For the secondary structure of the protein, the SPOMA (accessed on 17 March 2026) online tool (https://npsa-prabi.ibcp.fr/cgi-bin/npsa_automat.pl?page=npsa%20_sopma.html) was employed for prediction and analysis. The tertiary structure of the protein was then predicted and analyzed via ExPASy (accessed on 17 March 2026) (https://www.expasy.org/).

Subsequent BLASTP homology searches of *Fcgrr1*, with a threshold of E ≤ 1 × 10^−15^, were conducted against the FungiDB database and the National Center for Biotechnology Information (NCBI) protein database (accessed on 17 March 2026) (https://www.ncbi.nlm.nih.gov/protein/). Genomic sequences and gene models for *Fusarium* species, including *F. anthophilum*, *F. subglutinans*, *F. bulbicole*, *F. mexicanum*, *F. agapanthi*, *F. globosum*, *F. fujikuroi*, *F. oxysporum*, *F. redolens*, *F. mundagurra*, *F. acutatum*, *F. verticillioides*, *F. coicis*, *F. napiforme*, *F. denticulatum*, and *F. pseudocircinatum*, were included in the comparative genomic analysis. Sequence alignments were conducted via BioEdit software 7.2.6.1, followed by the elucidation of phylogenetic relationships through the application of the neighbor-joining algorithm implemented in MEGA version 6. Default parameters were employed, and the robustness of the phylogenetic trees was assessed through 1000 bootstrap replications to ensure their reliability.

### 2.4. Determination of the FcLrr1 Expression Level of Target Genes During the Infection Stage

To evaluate the expression levels of the four target genes, RT–qPCR was performed with the specific primers outlined in Table 1. The genomic DNA fragment coding for the β-actin gene (one copy in the genome) was selected as a control. The primers used for the β-actin gene were qatF and qatR (Table 1). All amplification products for the targets were between 100 and 200 bp in length. The infection stage samples were the seedlings collected at 12 h postinoculation, 36 h, and 3, 5, and 7 dpi. The concentration of cDNA was adjusted to 500 μg/μL, as determined by DNA fluorometry. qPCR was performed via TB Green Premix Ex Taq II (Tli RNaseH Plus) (TaKaRa, Dalian, China). The qPCR system consisted of the following components: 12.5 µL of 2× RT–PCR buffer (containing SYBR Green and Premix Ex Taq from TaKaRa), 0.5 µL of both the forward and reverse primers (each at 20 µM), 2 µL of genomic DNA, and 9.5 µL of ddH_2_O. The quantitative real-time PCR was carried out on a Mastercycler (Eppendorf, Carlsbad, CA, USA) with the following thermal cycling conditions: initial denaturation at 95 °C for 2 min, followed by 40 cycles of 95 °C for 10 s and 60 °C for 20 s, ending with a melting curve step. Each group of qPCR was repeated three times, and the average value was calculated. The data were analyzed via the 2^−ΔΔCt^ method [41].

### 2.5. Generation of FcLRR1 Deletion Construct and Complementation Construct

To achieve gene knockout, homologous recombination was utilized. Taking the CDS region of the *FcLRR1* gene as the center, approximately 1000 bp upstream and downstream sequences were selected as homologous arms to design primers to amplify homologous arms from A015 genomic DNA. In parallel, primers were created to amplify the hygromycin phosphotransferase gene (hph) from an earlier knockout mutant. The primer sequences were shown in Table 1. Ligation of the T-vector PMD19 with the homologous arms and hph gene fragment was performed via the Uniclone One Step Seamless Cloning Kit (SC612) (Genes and Biotech Co., Ltd., Nanjing, China). The ligation reactions included 5 μL of T-vector PMD19, 1 μL of each homologous arm fragment, 1.5 μL of the hph gene fragment, and 5 μL of 2× Uniclone Seamless Cloning Mix and were incubated at 50 °C for 30 min. The resulting recombinant plasmid was transformed into Escherichia coli DH5α competent cells (TransGen Biotech, Beijing, China). The plasmid was gently mixed with 50 μL of DH5α cells and then incubated on ice for 25 min. A heat shock at 42 °C for 30 s was followed by a 2 min incubation on ice. Afterward, 500 μL of LB liquid medium was added, and the mixture was shaken at 200 rpm at 37 °C for 1 h. Subsequently, 100 μL of the culture was spread onto LB plates containing 1 μL/mL carbenicillin overnight incubation, followed by colony PCR to screen for positive transformants. The positive transformants will be subjected to sequencing, and those with confirmed correct results will serve as templates for amplifying the fusion fragments containing the homologous arms and the hph gene, which will be utilized in the subsequent gene knockout procedures.

To construct the complementation vector for the *FcLRR1* gene, the PYF11 vector was first linearized by restriction digestion with the *Xho*I enzyme. Subsequently, specific primers with homologous sequences were designed to ensure overlap with the flanking regions of the PYF11 vector, and the *Fcgrr1* gene was amplified from the genomic DNA. The linearized PYF11 vector and the grr1 homology arms were then assembled via the Uniclone One Step Seamless Cloning Kit on the basis of homologous recombination. The resulting plasmids were transformed into *E. coli* DH5α competent cells for propagation and subsequent verification via Sanger sequencing to confirm the successful integration of the grr1 gene into the PYF11 vector. The transformation method was as described above.

### 2.6. Transformation of the Wild-Type and Mutant Strains

The mycelia of *F. circinatum* after 5 d were cut into 25 pieces of mycelial plugs (2 × 2 mm^2^) via a sterile scalpel and cultured in 50 mL of PDB at 28 °C with shaking at 160 rpm for 24 h to obtain sufficient conidia. The conidia were collected via sterilized filter cloth and centrifuged at 4000 rpm for 5 min. The conidia were resuspended in sterile water, and 50 mL of YEPD liquid medium was added at 28 °C with shaking at 90 rpm for 12 h. The germinated conidial hyphae were filtered through sterile filter cloth and subsequently incubated in enzymatic hydrolysate containing 5 mg/mL lysing enzymes from *Trichoderma harzianum* (Sigma Cat# 1412), 12.5 mg/mL Driselase from *Basdiomycetes* sp. (Sigma Cat# D9515), 7.5 mg/mL Snailase (Sigma Cat# C6137) and 20 mL of 1.2 M KCl solution. Enzymatic hydrolysis was conducted at 30 °C in a shaking incubator set at 65 rpm for 180 min. The quantity and quality of the protoplasts were assessed via a hemocytometer. This experiment was conducted in triplicate.

After digestion on a 30 °C table, the protoplasts were filtered through sterile filter cloth and centrifuged at 3000 rpm for 5 min, after which the supernatant was discarded. The pelleted protoplasts were resuspended in 10 mL of 1.2 M KCl and subjected to a second centrifugation at 3500 rpm for 5 min at 4 °C. Afterward, 1 mL of STC solution was added to each sample for resuspension. Centrifugation was repeated under the same conditions to collect the protoplasts, which were subsequently resuspended in 1 mL of STC solution at a concentration of 2–3 × 10^7^ cells/mL. Then, 10 µg of plasmid was added to 150 μL of protoplasts. The protoplasts were then gently mixed with 100 μL of PTC solution and placed on ice for 30 min to promote transformation. Subsequently, 1 mL of SPTC solution was added to the protoplast mixture, which was then allowed to rest at room temperature for 20 min. The mixture was subsequently transferred into 10 mL of TB3 liquid medium and incubated in the dark at 28 °C and 90 rpm for 12 h. Following this incubation period in TB3 liquid medium, the mixture was blended with TB3 agar medium containing 100 μg/mL hygromycin B and poured into plates. Finally, each plate was overlaid with 10–12 mL of TB3 containing 200 μg/mL Hyg, which was then inverted and maintained at 28 °C in the dark for an additional 4 d. Under these conditions, transformants capable of growing in the presence of hygromycin were expected to develop.

### 2.7. Identification of Gene-Deleted Mutants and Complementation Strains

The effective deletion of the target gene in individual transformants was determined by PCR. According to the protocol outlined previously, each transformant was subjected to total DNA extractionand used for PCR with the primer pair FcLRRs-In-F/R, which amplifies part of *FcLRR1* (Table 1). PCR was performed under the following conditions: 5 min at 94 °C, followed by 33 cycles of 15 s at 94 °C, 15 s at 58 °C and 30 s at 72 °C; the final elongation step consisted of 10 min at 72 °C. PCR amplification verification was also performed via upstream and downstream outer primers (FcLRRs-Out-F/Hyg-R, Hyg-F/FcLRRs-Out-R) and internal Hyg primers (Hyg-F/Hyg-R). The extension time in the amplification system for the two pairs of outer arm primers was 5 min at 94 °C, followed by 34 cycles of 15 s at 94 °C, 15 s at 58 °C and 1.5 min at 72 °C; the last step was extended for 10 min at 72 °C. Specific primer information is listed in Table 1. The amplified products were then subjected to gel electrophoresis. If no internal band of the target gene was amplified and both outer arm primer pairs produced bands, the transformant was highly likely a positive knockout mutant.

To further validate the successful knockout of the target gene, qRT–PCR analysis was performed via the ΔΔCt method. Briefly, transformants obtained from the initial screening and the wild-type strain A015-1 were cultured for 5 d. Total mRNA was extracted from both groups and immediately reverse-transcribed into cDNA. The cDNA was stored at −20 °C, while the residual mRNA was preserved at −80 °C. For qRT–PCR, SYBR Green-based assays were conducted on an ABI 7500 system with gene-specific primer pairs (Q-*FcLRR1*-F/R, Q-FcLRR2-F/R, Q-FcLRR3-F/R, and Q-FcLRR4-F/R) and an internal reference primer set (Q-Actin-F/R). All reactions were normalized prior to analysis. The relative expression levels of the target genes were calculated via the ΔΔCt method, where the fold change was determined via the 2^−ΔΔCt^ method. Transformants exhibiting negligible target gene expression compared with the wild-type control (ΔΔCt approaching zero) were identified as successful knockout candidates.

To confirm successful genetic complementation, PCR validation was performed via internal primers and G418 fragment-specific primers (G418-F/R). Successful complementation was defined by the amplification of specific bands from both primer sets, coupled with sequencing verification of correct integration. The validated transformants were subsequently subcultured for three generations on hygromycin B (HPH)-containing PDA medium to ensure genetic stability. Only transformants retaining stable complementation after revalidation were selected for further experiments.

### 2.8. Subcellular Localization Observation

The PYF11 plasmid was digested with *Xho*1 fast-digest enzyme in a 50 μL reaction mixture containing 20 μL of PYF11 plasmid, 5 μL of Xho1 fast-digest enzyme, and 25 μL of ddH_2_O. The digestion was performed at 37 °C for 1 h. After digestion, the products were recovered for subsequent experiments. A mixture of 7 μL of the target gene fragment, 2 μL of linearized PYF11 vector, and 9 μL of salmon sperm carrier DNA was transformed into XK125 competent yeast cells. The cells were incubated at 30 °C for 30 min with shaking, incubated at 42 °C for 20 min with shaking, and then chilled on ice for 5 min to complete the transformation. The cells were centrifuged at 5000 rpm for 1 min, the supernatant was discarded, and the cells were resuspended in 100 μL of sterile water before being spread onto a selective medium plate lacking one nutrient. The plates were incubated at 30 °C for 2 d. Yeast single colonies grown on selective media were subjected to PCR verification. Positive transformants were selected and cultivated overnight in YPDA liquid media with shaking. The cells were then collected by centrifugation at 10,000 rpm, and the yeast plasmids were extracted. Five microliters of the yeast plasmid was introduced into competent *E. coli* cells to screen for positive transformants, which were subsequently sent for sequencing. The sequencing results were compared with the target gene sequence. Plasmids from *E. coli* positive transformants with correct sequences were used for transformation experiments, following the procedure described in Section 2.4. After the transformants were grown, they were initially screened via a fluorescence microscope, and those with higher fluorescence intensity were selected for three generations of single-spore purification. The purified transformants were observed under a microscope for fluorescence, and their DNA was extracted for verification via internal primers for GFP and the target gene, followed by sequencing. The successful construction of the FcLRR1-GFP fusion strain was confirmed via sequence alignment. A 10 μL aliquot of spore suspension (concentration: 1 × 10^4^ spores/mL) was dripped onto a hydrophobic glass slide and incubated in darkness at 25 °C for 24 h for subsequent fluorescence observation.

### 2.9. Phenotypic Assay of the ∆Fcgrr1 Strain and the Wild-Type Strain

#### 2.9.1. Morphological Traits of the Mutant and Wild-Type Strains

The colony morphologies of wild-type A015, the knockout mutant Δ*FcLRR*, and the complementary mutant FcLRR1-c were examined. After growing on PDA plates for 5 d, agar blocks colonized with mycelia were punched out along the colony edges via a sterile 5 mm diameter puncher. These agar blocks were then placed separately on 70 mm PDA Petri dishes and cultivated at 28 °C under a 12/12 h light/dark cycle for 5 d to observe colony growth. On the 5th day, the diameters of the fungal colonies were measured via a cross method centered on the mycelial block. Six mycelial plugs were cultivated in PDA liquid at 28 °C and 160 rpm for 24 h. The mycelia were subsequently collected and dried in an oven at 55 °C for 60 min to remove moisture, after which their dry weights were measured via an analytical balance. Each experiment was repeated independently three times, and the average values were calculated.

#### 2.9.2. Conidial Assay

To quantify conidial production, the wild-type strain A015-1 and all the knockout mutant strains were cultured on PDA plates at 25 °C in the dark for 3 d. Five mycelial plugs (5 mm diameter) were excised from the colony margins via a sterile fungal disc puncher and inoculated into 250 mL Erlenmeyer flasks containing 50 mL of MBM liquid medium. The cultures were incubated at 28 °C with agitation (160 rpm) for 24 h to induce sporulation. For spore enumeration, 10 μL aliquots of the conidial suspension were loaded onto a hemocytometer and examined under an optical microscope.

To determine the conidial germination rate, conidial suspensions were prepared according to the aforementioned protocol. One milliliter of the wild-type or knockout mutant suspensions was transferred to 2 mL sterile centrifuge tubes. After centrifugation at 5000 rpm for 5 min at room temperature, the supernatants were discarded, and the pellets were collected. The pellets were subsequently resuspended in 1 mL of sterile distilled water and incubated at 25 °C. At 4 h, 6 h, and 12 h postincubation, 10 μL of the suspension was placed on glass slides for microscopic observation under a microscope. Germination rates was calculated by quantifying the number of germinated conidia among ≥200 randomly selected conidia at each time point.

Conidial germination was evaluated in the wild-type, knockout, and complemented mutant strains. To this end, conidial suspensions (1 mL) of the wild-type strains A015-1 and Δ*FcLRR1* were transferred to sterile 12-well culture plates (Corning 3513, Beijing, China) and incubated in darkness at 25 °C at 80 rpm. At 12 h, 24 h, and 48 h postinoculation, 10 μL aliquots were sampled and mounted on glass slides. The germ tube lengths (measured from the spore apex to the hyphal tip) of 20 randomly selected germinated conidia per field were quantified via a calibrated ocular micrometer on a Zeiss microscope (Carl Zeiss AG, Oberkochen, Germany).

#### 2.9.3. Stress Adaptation Assay

To assess the differential sensitivity of *F. circinatum* and its mutant strains under various stress conditions, 5 mm mycelial plugs from each strain were inoculated onto PDA plates supplemented with 1 M sorbitol, 1 M NaCl, 6 M H_2_O_2_, 600 μg/mL Congo red and 0.01% SDS. Following inoculation, the plates were incubated at 28 °C in darkness for 5 d. Colony diameters were measured as outlined in previous protocols, and each treatment was performed in triplicate.

### 2.10. Inoculation of P. elliottii Seedlings with F. circinatum

To evaluate the impact of *FcLRR1* knockout on pathogenicity, lesion length on seedling stems was measured following inoculation. For 2-week-old seedlings, conidial suspensions (10^5^ spores/mL) of the wild-type strain A015-1 and the *FcLRR1* knockout mutant were prepared. Each strain was inoculated onto 20 seedlings, with lesion lengths recorded at 5 and 7 d. The experiment was performed in triplicate. Disease severity was classified into five grades (0 = healthy, no necrosis; 1 = localized necrosis at the inoculation site; 2 = necrosis extending 0.5–1 cm; 3 = necrosis > 1 cm; 4 = necrosis > 2 cm). The disease index (*DI*) and disease incidence were calculated as follows:

(1) $$DI=\frac{\sum (\mathrm{Number}\;\mathrm{of}\;\mathrm{plants}\;\mathrm{per}\;\mathrm{grade}\;\times \;\mathrm{Grade}\;\mathrm{value})\;}{\mathrm{Total}\;\mathrm{piants}\;\mathrm{suryed}\;\times \;\mathrm{Highest}\;\mathrm{grade}\;\mathrm{value}}\times 100$$

(2) $$\mathrm{Disease}\;\mathrm{incidence}\;(\%)=\frac{\mathrm{Number}\;\mathrm{of}\;\mathrm{infected}\;\mathrm{piants}}{\mathrm{Total}\;\mathrm{piants}\;\mathrm{surveyed}}\times 100$$

For pathogenicity evaluation of 1-month-old seedlings, the apical 0.5 cm of each seedling was excised aseptically via a sterile scalpel. A 10 μL aliquot of conidial suspension (10^5^ spores/mL) from either the wild-type strain A015-1 or the *FcLRR1* knockout mutant *FcLRR1*-49 was inoculated onto the wounded apex. Three seedlings were inoculated per strain, with control seedlings receiving 10 μL of sterile ddH_2_O. Postinoculation, the seedlings were incubated in a growth chamber at 28 °C under a 12 h light/dark cycle. To maintain humidity, the chamber was misted with sterile water every 24 h. After 20 d, disease progression was assessed by measuring the length of necrotic tissue. The experiment was conducted in triplicate.

### 2.11. Statistical Analysis

Statistical analysis of the data was performed via Duncan’s multiple range test. Significant differences compared with the control group (*p* < 0.05) are marked with asterisks in the figures and tables. Different letters denote statistically significant differences between treatment groups at *p* < 0.05.

### 2.12. Transcriptome Analysis

The raw sequencing data were initially processed via Trimmomatic v0.39 to remove low-quality reads, reads with more than 10% unidentified nucleotides (N), and adapter sequences. The filtered data were subsequently subjected to quality control assessment via FastQC to obtain clean data, which were then assembled into transcripts via Trinity (v2.14.0), from which the longest transcript per gene (unigene) was selected as the functional gene representative. These unigenes were used as the reference for read alignment with HISAT2, and gene expression levels were quantified via RSEM, with TPM (transcripts per million) adopted as the expression metric. The functional annotation of the unigenes was performed by alignment against the GO and KEGG databases. Differential gene expression analysis was carried out via DESeq2, with significantly differentially expressed genes identified on the basis of a false discovery rate (FDR) and a fold change threshold.

### 2.13. Yeast Two-Hybrid Assay

To identify proteins in *F. circinatum* that interact with the bait protein FcLRR1 with high binding energy, we employed an integrated bioinformatics pipeline. First, three-dimensional structural models of all genome-encoded proteins were generated via OmegaFold. A comprehensive workflow was subsequently established by integrating RFdiffusion, FoldSeek, HDock (v1.1), and AlphaFold3 (v3.0.1), enabling systematic identification of binding sites and meticulous selection of candidate interacting proteins.

On the basis of our bioinformatic predictions, ALG-11—a protein involved in cell wall synthesis—was selected for experimental validation of its potential interaction with FcLRR1. The bait vector pGBKT7-FcLRR1 and prey vector pGADT7-ALG-11 were constructed and supplied by Nanjing Ruiyuan Biotechnology Co., Ltd (Nanjing, China).

To rule out autoactivation by the bait protein, pGBKT7-FcLRR1 was cotransformed with the empty pGADT7 vector into the yeast strain AH109. Eight single colonies from the cotransformations were randomly selected for PCR verification and strain preservation. Three colonies yielding correct PCR results were then randomly chosen, cultured to OD_600_ = 0.2, and spotted onto SD/-Trp/-Leu (SD-TL), SD/-Trp/-Leu/-His (SD-TLH), SD/-Trp/-Leu/-His/-Ade (SD-TLHA), and SD-TLHA supplemented with X-α-gal plates. All plates were incubated at 30 °C for 3–5 d until colony growth was observed.

The pGBKT7-FcLRR1 with pGADT7-ALG-11, pGBKT7 (empty vector) with pGADT7-ALG-11, pGBKT7-FcLRR1 with pGADT7 (empty vector), and positive and negative control plasmids were separately transformed into yeast cells. For each transformation, colonies were picked and resuspended in 100 µL of sterile water, followed by vigorous vortexing to achieve homogeneous suspensions. Subsequently, 5 µL of each suspension was spotted onto SD/-Trp/-Leu (SD-TL), SD/-Trp/-Leu/-His (SD-TLH), SD/-Trp/-Leu/-His/-Ade (SD-TLHA), and SD-TLHA supplemented with X-α-gal plates. All plates were incubated inverted at 30 °C for 3–5 d until colony growth occurred.

## 3. Results

### 3.1. Identification of the FcLRR1 Gene in F. circinatum

In *F. circinatum*, a total of 13 proteins containing leucine-rich repeat (LRR) domains were identified through bioinformatics analysis. These proteins are designated sequentially as FcLRR1–FcLRR13, with their corresponding amino acid counts and the number of LRR domains detailed in Table 2.

This study focused on FcLRR1, and sequence analysis revealed that FcLRR1 consisted of multiple β-α repeat units (LRR regions), an α-helix-dominated functional domain (F-Box), and unstructured coils via the SOPMA online tool (Figure S1A). Further analysis via the SMART tool predicted 12 consecutive LRR domains spanning residues 165–497, along with a single F-Box domain located at residues 74–122 (Figure S1A). Structural analysis of the three-dimensional structural model revealed distinct features of the LRR domain, with a central conserved region consisting of 12 tandem LRR motifs, which likely facilitates core functions, including ligand recognition and protein interactions. The presence of an F-Box domain, which is typically associated with binding interfaces for the SCF complex, suggests a potential role in ubiquitin-mediated protein degradation pathways.

To assess the evolutionary relationships of FcLRR1, a BLASTP search (E value ≤ 1 × 10^−50^) was conducted against *Fusarium* and related genera via the FungiDB genome database (version 42). The resulting interspecific phylogenetic tree revealed broad conservation of FcLRR1 homologs across various *Fusarium* species. Notably, FcLRR1 clustered independently within a distinct branch, demonstrating the closest affinity to *F. mexicanum*. This clade was resolved with strong support, which is consistent with the evolutionary divergence observed among Fusarium species complexes (Figure S2).

### 3.2. Gene Expression Model and Location of FcLRR1

**Figure 1:**
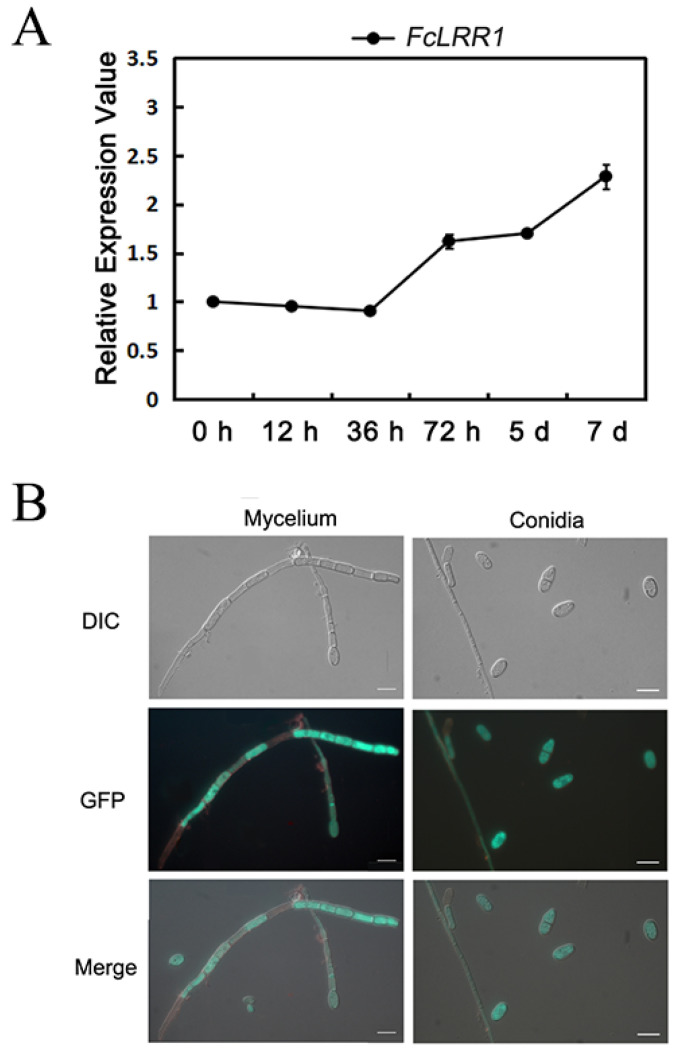
Transcription levels and subcellular localization of the FcLRR1 gene (**A**): Transcriptional levels of the *FcLRR1* gene at different stages in the host (two-week-old *P. elliottii* seedlings) infected with *F. circinatum.* (**B**): Subcellular localization of the FcLRR1 protein. Bar = 10 μm. Upper: Hyphae and Spores DIC Image; Middle: Hyphae and Spores GFP Image; Lower: Hyphae and Spores Merge.

To investigate the temporal expression dynamics of *FcLRR1* during infection, qRT–PCR analysis was performed at five time points (12 h, 36 h, 3 d, 5 d, and 7 d post-inoculation) following the inoculation of slash pine seedlings. The results revealed that *FcLRR1* expression remained stable during the early infection phase (12–36 h) but progressively increased thereafter, peaking at 72 h, with expression levels approximately 2.5-fold greater than those of the control (Figure 1A). These findings suggest that *FcLRR1* may be involved in the pathogenicity of *F. circinatum*.

To further elucidate the subcellular localization of FcLRR1, the *FcLRR1* gene was cloned, fused with GFP, and transformed into the wild-type strain A015-1. Transformants stably expressing the *FcLRR1*-GFP fusion gene were selected on the basis of G418 resistance, fluorescence intensity, and unchanged colony morphology. Fluorescence microscopy revealed uniform, high-intensity GFP signals in the cytoplasmic matrix of both hyphae and conidia, with no nuclear localization observed (Figure 1B).

### 3.3. Generation of the FcLRR1 Deletion Mutant and Complementation Strains

To examine the cellular function of *FcLRR1*, a gene replacement construct was constructed and used for the transformation of wild-type A015-1 protoplasts to obtain the ∆*FcLRR1* mutants. Five putative transformants exhibiting typical growth were successfully obtained on PDA supplemented with 100 μg/mL hygromycin B. PCR analysis confirmed the absence of the *FcLRR1* gene in all the transformants while successfully amplifying the hygromycin resistance cassette and its flanking regions. This finding indicated that the *FcLRR1* gene was replaced by *HPH* in the A015-1 genome (Figure S3A). qRT–PCR analysis revealed nearly complete loss of *FcLRR1* transcription in the knockout mutants ∆*FcLRR1*-20, ∆*FcLRR1*-25, and ∆*FcLRR1*-49. The ∆*FcLRR1*-49 mutant was randomly chosen for further phenotypic characterization (Figure S3B). For genetic complementation, PCR screening of colonies grown on G418-containing PDA identified more than 10 candidate transformants, of which only two retained the complemented *FcLRR1* (Figure S3). One of the complemented strains, designated *FcLRR1*-c, was chosen for further phenotypic analysis.

### 3.4. Deletion of FcLRR1 Affects the Morphology and Growth of F. circinatum

**Figure 2:**
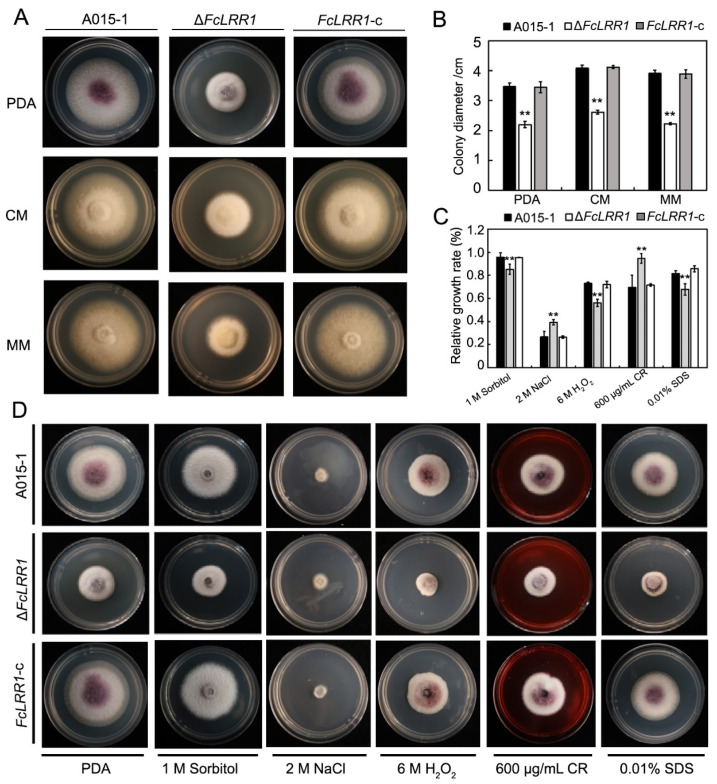
The growth and stress phenotypes of the wild-type strain A015-1, the knockout mutant ΔFcLRR1, and the complemented strain FcLRR1-c. (**A**) Growth plate images of the wild-type strain A015-1 and the knockout mutant Δ*FcLRR1* on PDA (Potato Dextrose Agar), CM (Complete medium), and MM (minimal medium) culture media. (**B**) Comparison of colony sizes between the wild-type and mutant strains. (**C**) Relative growth of the wild-type and the mutant strains on PDA culture media containing different stresses (relative to colony growth without stress). (**D**) Growth plate images of the wild-type and mutant strains on PDA culture media subjected to different stresses. (**: *p* < 0.01).

To evaluate the effects of *FcLRR1* deletion on fungal growth, wild-type A015-1 and the Δ*FcLRR1* mutant were inoculated on PDA, complete medium (CM), or minimal medium (MM) and then incubated at 25 °C for 5 d. The colony diameters were measured via the cross-diameter method. Compared with the wild-type strain, the Δ*FcLRR1* mutant exhibited significantly reduced growth across PDA, CM and MM media (Figure 2A,B). Notably, the mutant presented fewer aerial hyphae and diminished pigment than did the wild type. These results suggest that *FcLRR1* disruption may impair hyphal development, nutrient utilization, and secondary metabolism in *F. circinatum*.

### 3.5. Disruption of FcLRR1 Affects Abiotic Stress Responses

To investigate the role of *FcLRR1* in the abiotic stress response of *F. circinatum*, the wild-type and Δ*FcLRR1* strains were cultured on PDA supplemented with 1 M sorbitol, 6 mM H_2_O_2_, or 0.01% sodium dodecyl sulfate (SDS). Compared with the wild type, the Δ*FcLRR1* mutant presented significantly reduced growth under H_2_O_2_, Sorbital and SDS conditions, suggesting that FcLRR1 is involved in regulating oxidative defense and cell wall integrity (Figure 2C,D). These findings underscored the complex regulatory role of FcLRR1 in balancing stress adaptation and metabolic trade-offs in *F. circinatum*.

### 3.6. FcLRR1 Is Involved in Conidium Production and Germination

**Figure 3:**
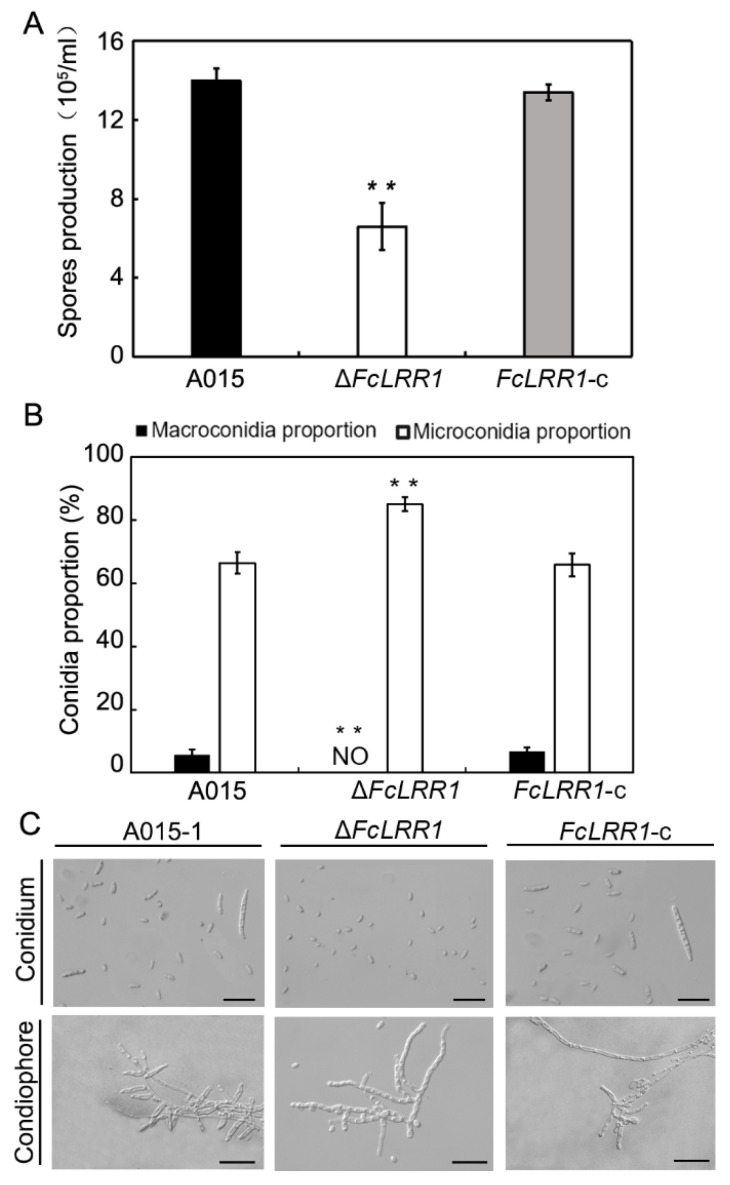
Conidia of the knockout mutant Δ*FcLRR1*1 and the wild-type strain A015-1. (**A**) Conidia production quantity. **: *p* < 0.01. (**B**) Conidial production composition. (**C**) Morphologies of conidia and conidiophores of the knockout mutant Δ*FcLRR1* and the wild-type strain A015-1. Bar = 20 μm.

To elucidate the role of *FcLRR1* in the conidiation and germination of *F. circinatum*, conidial production and morphological composition were compared between the wild-type A015-1 and the Δ*FcLRR1* mutant. The Δ*FcLRR1* mutant presented a significant reduction in conidial yield. After 24 h of shaking culture in MBM, the wild-type strain produced approximately 13.5 × 10^5^ conidia/mL, whereas the Δ*FcLRR1* mutant yielded only 5 × 10^5^ conidia/mL (Figure 3A). Morphological analysis revealed distinct differences in conidial composition. The wild-type A015-1 typically produced two main conidial morphotypes: microconidia (66.4 ± 3.36%) and macroconidia (5.8 ± 1.48%). In contrast, the Δ*FcLRR1* mutant completely lost the ability to produce macroconidia, resulting in an increase in the proportion of microconidia to 85 ± 2.24% (Figure 3B). Furthermore, abnormal conidiophore morphology was observed in the mutant (Figure 3C).

**Figure 4:**
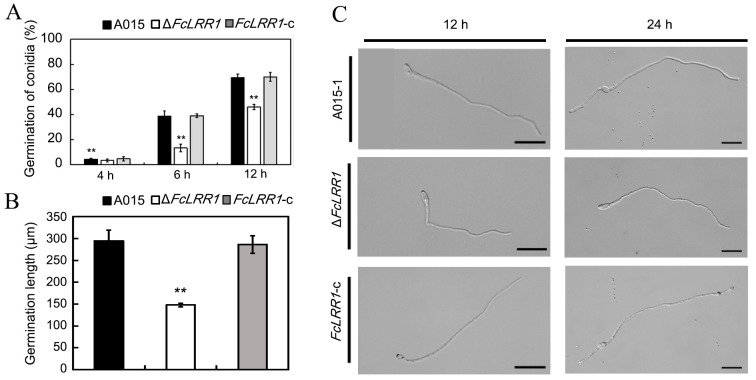
Conidium germination rates of the knockout mutant Δ*FcLRR1* and the wild-type strain A015-1. (**A**) Conidium germination rates of the ΔFcLRR1 knockout mutants. **: *p* < 0.01. (**B**) Conidium germination conditions of Δ*FcLRR1* at 12 h and 24 h. **: *p* < 0.01. (**C**) Conidium germination lengths of Δ*FcLRR1* at 48 h. Bar = 20 μm.

The germination rate was assessed at 4 h, 6 h, and 12 h postincubation. Compared with the wild type, the ΔFcLRR1 mutant presented a significantly lower germination rate throughout the 4–12 h period (Figure 4A). Furthermore, germ tube elongation was markedly delayed in the mutant at 12 h, 24 h and 48 h, with the germ tubes exhibiting stunted growth and reduced branching (Figure 4B,C). These results demonstrate that FcLRR1 was critical for regulating conidial development, morphological diversification, and germination efficiency in *F. circinatum*, underscoring its essential role in fungal propagation and pathogenesis.

### 3.7. FcLRR1 Affects the Pathogenicity of F. circinatum

**Figure 5:**
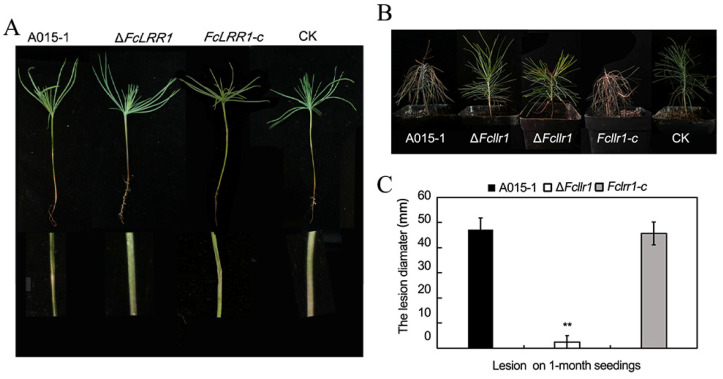
Pathogenicity of the wild-type strain A015-1 and the knockout mutant Δ*FcLRR1* on *P. elliottii.* (**A**) Pathogenicity results of the wild-type strain and the knockout mutant ΔFcLRR1 after infecting two-week-old slash pine seedlings. Upper: Overall disease incidence of seedlings;Lower: Diseased tissues; CK: Blank control; Bars = 1 cm. (**B**) Pathogenicity results of the wild-type strain and the knockout mutant ΔFcLRR1 after infecting one-month-old *P. elliottii* seedlings. From left to right: Disease occurrence of A015-1, ΔFcLRR1, FcLRR1-c, and CK; CK represents the blank control. (**C**) Mean lesion lengths caused by the wild-type strain and the knockout mutant ΔFcLRR1 after infecting one-month-old *P. elliottii* seedlings. CK: blank control; ΔFcLRR1 has two states: no disease and mild disease. **: *p* < 0.01.

Pathogenicity assays were conducted on 2-week-old and 1-month-old *P. elliottii* seedlings, as well as on slash pine, to evaluate virulence attenuation in the ΔFcLRR1 mutant. For 2-week-old seedlings inoculated with 10 μL of conidial suspension (1 × 10^5^ conidia/mL), disease progression in the ΔFcLRR1 strain was significantly delayed and less severe than that in the wild-type A015-1 strain (Figure 5A). At 5 days post- infection (dpi), the disease index (DI) of wild-type-infected seedlings reached 70 ± 4.25, with only 10% remaining asymptomatic. In contrast, the ΔFcLRR1 strain presented a DI of 12.5 ± 0.5 and a 65% asymptomatic rate. By 7 dpi, the wild-type DI further increased to 83.75 ± 5 (with 10% asymptomatic), whereas the mutant DI rose to 53.75 ± 3.75 but maintained a significantly higher asymptomatic rate (30% compared with 10% in the wild type) (Table 3). These results indicated that the deletion of *FcLRR1* not only severely compromised pathogen virulence but also delayed the onset of disease.

In 1-month-old seedlings of *P. elliottii* inoculated with the same conidium suspension, the lesion lengths measured at 20 d revealed significant differences: the wild-type A015-1 caused lesions averaging 47.30 ± 4.54 mm, whereas the ΔFcLRR1 mutant induced negligible lesions measuring only 2.43 ± 2.61 mm. This observation demonstrated the inability of the mutant to effectively colonize older host tissues (Figure 5B,C). Collectively, these findings underscored the critical role of FcLRR1 in mediating the pathogenicity of *F. circinatum* across the developmental stages of slash pine, as its absence renders the fungus largely avirulent in mature seedlings.

### 3.8. Data Quality Assessment and Differential Expression Analysis

**Figure 6:**
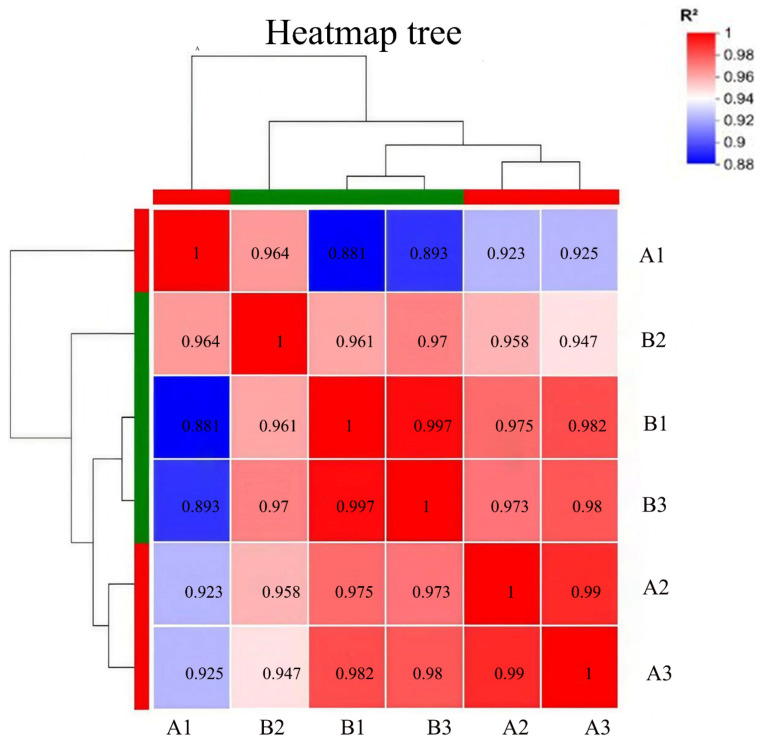
Heat map of the correlation between samples A1, A2, and A3 are wild-type strains; B1, B2, and B3 are FcLRR1 knockout mutant strains. Furthermore, compared with the wild-type strain, the mutant strain presented 555 upregulated and 612 downregulated differentially expressed genes, which are implicated in the pathogenicity mechanisms of the fungus (Figure 7).

Transcriptome sequencing of six samples yielded a total of 38.28 Gb of clean data. All samples exhibited high sequencing quality, with Q20 and Q30 scores exceeding 98.2% and 94.79%, respectively, and GC contents ranging from 52.84% to 53.19% (Table 4), indicating data reliability for subsequent bioinformatic analyses. Clean reads from each sample were aligned to the reference genome of *F. circinatum*, achieving mapping rates between 96.73% and 98.35%. The correlation coefficients among the biological replicates ranged from 0.923 to 0.99, confirming high reproducibility across the replicates (Figure 6). Furthermore, compared with the wild-type strain, the mutant strain presented 555 upregulated and 612 downregulated differentially expressed genes, which are implicated in the pathogenicity mechanisms of the fungus (Figure 6).

### 3.9. KEGG Pathway Enrichment Analysis of Differentially Expressed Genes

A total of 1137 differentially expressed genes (DEGs) were mapped to the KEGG database and annotated to 82 metabolic pathways across seven major categories. The highly enriched pathways included cyanoamino acid metabolism, starch and sucrose metabolism, ABC transporters, and amino sugar and nucleotide sugar metabolism (Figure 8). Notably, DEGs involved in the starch and sucrose metabolism pathways were significantly upregulated, suggesting that the overexpression of a specific gene may increase glycosyl hydrolase or glycosyltransferase activity, thereby improving carbon source utilization efficiency and providing energy for the infection process. The significant enrichment of ABC transporters implies their potential role in mediating the transmembrane transport of small molecules. Additionally, the amino sugar and nucleotide sugar metabolism pathways may contribute to the biosynthesis of cell wall components such as chitin and glucan by regulating the synthesis of precursors such as UDP-glucose or GDP-mannose, thereby maintaining the structural integrity of the pathogen cell wall. This mechanism was highly consistent with the previously observed biological function of the Fclrr1 gene, which affects conidial germination and mycelial expansion in the pine resin canker pathogen.

### 3.10. Structural Modeling and Molecular Docking of the FcLRR1 Protein

Following sequence filtering and OmegaFold modeling, a three-dimensional structural library comprising 12,071 proteins from the pine resin canker pathogen was successfully constructed. The three-dimensional structure of FcLRR1 was retrieved from the resulting database, revealing a 472-amino-acid protein containing 12 tandem leucine-rich repeat (LRR) motifs (Figure S4). Through an integrated workflow involving binder design by RF diffusion, structural alignment by FoldSeek, refined screening with HDock (v1.1), and interaction confidence assessment by AlphaFold3, the ALG-11 protein was identified as a high-confidence candidate with extensive predicted interfacial complementarity to the bait protein. The molecular docking results were visualized via PyMOL 3.1 (Figure S5).

### 3.11. Yeast Two-Hybrid Autoactivation Assay

The positive controls grew normally on SD/-Trp/-Leu (SD-TL), SD/-Trp/-Leu/-His (SD-TLH), SD/-Trp/-Leu/-His/-Ade (SD-TLHA), and SD-TLHA supplemented with X-α-gal plates and produced blue colonies on X-α-gal-containing media. In contrast, the negative control only grew on SD-TL plates and failed to grow on SD-TLH, SD-TLHA, or SD-TLHA + X-α-gal plates. The bait control (pGBKT7-FcLRR1 + pGADT7) presented a growth pattern consistent with that of the negative control, confirming the absence of autoactivation activity in the pGBKT7-FcLRR1 construct and verifying its suitability for further yeast two-hybrid interaction assays.

### 3.12. Yeast Two-Hybrid (Y2H) Assay

In yeast two-hybrid (Y2H) assays, the interaction between pGBKT7-FcLRR1 and pGADT7-ALG11 was investigated. The *FcLRR1* gene on the pGBKT7 vector and the *ALG11* gene on the pGADT7 vector were cotransformed into the yeast strain AH109. The analysis included the experimental groups (pGBKT7- FcLRR1 and pGADT7-ALG11), the control groups (pGBKT7 and pGADT7-ALG11, pGBKT7-FcLRR1 and pGADT7), the positive control groups (pGADT7-largeT + pGBKT7-p53), and the negative control groups (pGADT7-largeT + pGBKT7-laminC). From each group, eight single colonies derived from cotransformed AH109 were selected at random for PCR and preservation. From the single colonies that yielded correct PCR results, three were randomly chosen from each group for spotting analysis.

**Figure 7:**
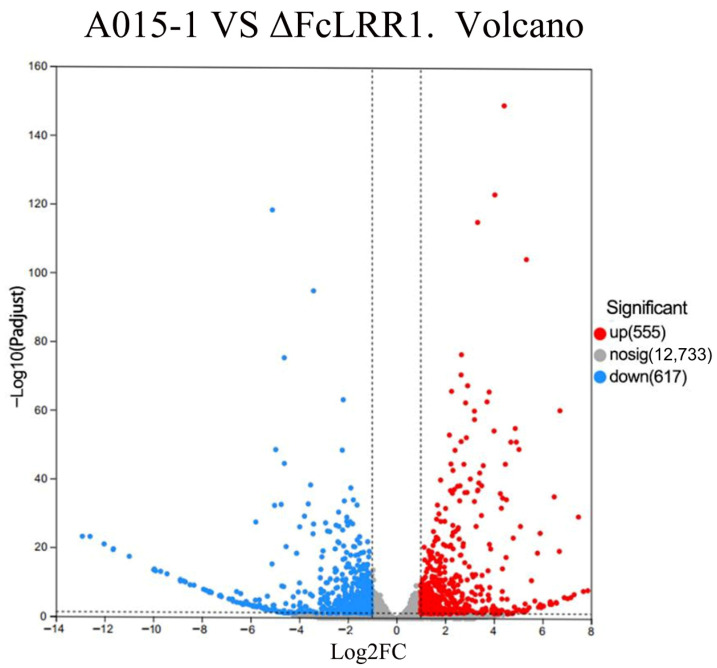
Venn diagram of differentially expressed genes between samples log2FC: The fold change in gene expression in the samples; −log10 (padjust): The statistical test value of the significance level of gene expression differences; The black dashed line: The threshold boundary for the selection criteria of differentially expressed genes.

**Figure 8:**
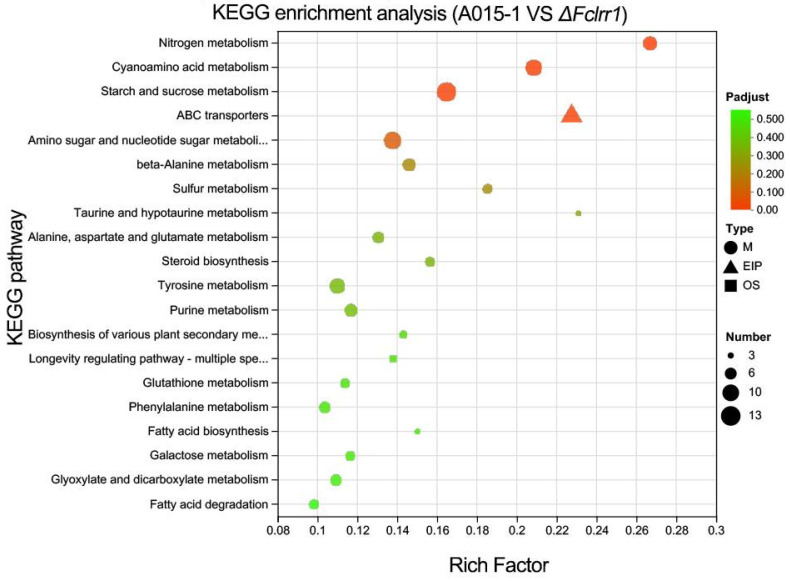
KEGG enrichment analysis of differentially expressed genes.

**Figure 9:**
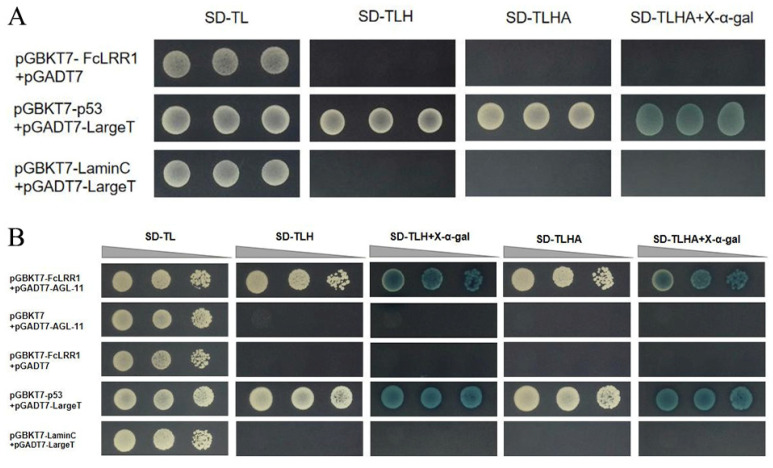
Yeast two-hybrid interaction verification. (**A**) Autologous activation verification of pGBKT7-FcLRR1. (**B**) Yeast two-hybrid interaction verification of pGBKT7-FcLRR1 and pGADT7-ALG-11.

The results revealed that both the experimental group (pGBKT7-FcLRR1 + pGADT7-ALG-11) and the positive control grew normally on SD/-Trp/-Leu (SD-TL), SD/-Trp/-Leu/-His (SD-TLH), SD-TLH supplemented with X-α-gal, SD/-Trp/-Leu/-His/-Ade (SD-TLHA), and SD-TLHA with X-α-gal plates. Additionally, they produced blue colonies on both SD-TLH + X-α-gal and SD-TLHA + X-α-gal media (Figure 7). In contrast, the bait control (pGBKT7 + pGADT7-ALG-11), prey control (pGBKT7-FcLRR1 + pGADT7), and negative control strains grew only on SD-TL plates and failed to grow on SD-TLH, SD-TLH + X-α-gal, SD-TLHA, and SD-TLHA + X-α-gal plates (Figure 9). These findings demonstrate a direct protein–protein interaction between FcLRR1 and ALG-11.

## 4. Discussion

The leucine-rich repeat (LRR) domain is a modular protein structure found across various biological kingdoms. It is characterized by repeats of 20–30 amino acid residues that form a horseshoe-shaped architecture consisting of alternating β-sheets and α-helices [42]. This structural motif provides LRR-containing proteins with remarkable ligand-binding capabilities, allowing them to play crucial roles in signal recognition, protein–protein interactions, and immune regulation [43]. Although LRR domains demonstrate significant functional diversification in plants, animals, and fungi, conserved core sequences (e.g., LxxLxLxxNxL) preserve their essential function as molecular recognition modules [44]. For example, the LRR domains of plant receptor-like kinases (LRR-RLKs) and animal Toll-like receptors (TLRs) exhibit mechanistic similarities in the recognition of pathogen-associated molecular patterns (PAMPs) [45,46].

In plants, LRR-containing proteins (LRR-RLKs and LRR-RLPs) are crucial for development and immunity. Structurally, their LRR domains form horseshoe-shaped folds that recognize diverse ligands, from peptide hormones to pathogen-associated molecular patterns (PAMPs) [47]. For example, *Arabidopsis* FLS2 binds to flg22, initiating immune signaling via the BAK1 and MAPK cascades [48,49]. Moreover, CLV1 and BRI1 mediate developmental processes [50]. The evolution of LRR-RLK subfamilies involves specialization in pathogen recognition and hormonal signaling, with LRR domains evolving rapidly [51]. In animals, LRR proteins are key to innate immunity, adhesion, and disease. TLRs detect PAMPs and trigger inflammatory responses. Toll-like receptors (TLRs) detect PAMPs, activating MyD88-dependent inflammatory responses through their TIR domains [45]. NOD-like receptors, such as NLRP3, sense cytosolic pathogens and assemble inflammasomes to activate caspase-1 [52]. In addition to their role in immunity, LRR proteins regulate development: *Drosophila* Toll proteins coordinate embryonic dorsoventral patterning through LRR-mediated ligand recognition [53], whereas human LRPs such as LRP5/6 regulate Wnt signaling, and their abnormal function contributes to cancer development [54]. Functional divergence is evident in disease contexts: somatic mutations in the LRR domains of TLRs have been linked to autoinflammatory disorders, whereas aberrant LRP6 signaling promotes tumor angiogenesis [55].

However, functional studies of LRR domain-containing proteins in fungi are limited, with the current knowledge being derived primarily from the model *Saccharomyces cerevisiae* and the human pathogen *Candida albicans*. In *S. cerevisiae*, LRR domain proteins are implicated in growth regulation, transcriptional regulation, and DNA damage repair. The first identified LRR protein in yeast, GRR1, contains an F-box domain and is essential for glucose repression signaling [56]. The most classical function of the LRR domain is its role as a substrate recognition module for SCF (Skp1-Cullin1-F-box) type E3 ubiquitin ligases. Early studies on glucose signaling mechanisms in *S. cerevisiae* revealed that the F-box protein Grr1, a component of the SCF complex, recruits substrate proteins through its LRR domain. Specifically, the LRR domain of Grr1 is responsible for recognizing specific substrates (such as G1 cyclins Cln1 and Cln2), while the adjacent F-box motif binds to the adapter protein Skp1 of the core complex [57]. This recruitment brings the substrate to the catalytic core for ubiquitination. This modular design, where the LRR domain is responsible for recognition and the F-box for linkage, represents one of the paradigms for substrate-specific recognition by the UPS. Subsequent studies have further confirmed that the substrate-binding function of the LRR domain within SCF complexes is conserved. For instance, a gain-of-function mutation affecting the surface charge of the concave face of the Grr1 LRR domain enhances its efficiency in degrading the substrate Mks1p, providing direct evidence that the spatial conformation of the LRR domain is critical for substrate recognition [58]. Similarly, the LRR protein CCR4 is required for the glucose-repressible transcription of alcohol dehydrogenase (ADH2) [59], and RAD1 and RAD7 are involved in nucleotide excision repair [60]. Notably, the LRR domain is essential for the Ras-dependent activation of adenylate cyclase [32]. In *C. albicans*, Xu et al. demonstrated that the adenylate cyclase Cyr1, which contains an LRR domain, binds to bacterial peptidoglycan (PGN)-derived molecules in serum. This interaction triggers the synthesis of cyclic adenosine monophosphate (cAMP) and promotes the yeast-to-hyphal transition, a critical virulence trait [33]. Bioinformatic analyses conducted by Soanes et al. revealed a substantial repertoire of LRR domain proteins in fungi; however, most of these proteins lack the canonical receptor domains typically observed in oomycetes and plants, suggesting divergent functional roles [31]. This structural distinction suggests that fungal LRR proteins may function through mechanisms that are unique and distinct from classical receptor-mediated pathways found in other eukaryotes. In this study, we identified 13 LRR-containing proteins in *F. circinatum*, among which FcLRR1 was highly conserved across *Fusarium* species. qRT–PCR analysis revealed sustained upregulation of *FcLRR1* expression during infection. Subcellular localization analysis revealed that FcLRR1 was uniformly distributed in the cytoplasmic matrix of both hyphae and conidia. These findings suggest that FcLRR1 may play pivotal roles in fungal growth, development, and metabolic processes, offering critical insights for further functional characterization of FcLRR1 in the pathogen’s lifecycle and virulence.

Members of the *Fusarium* genus, which are significant plant pathogens and saprophytic fungi, have been a central focus in the field of fungal molecular pathology, particularly concerning their developmental processes, conidiation, and virulence. In *Fusarium* species, the MAPK and cAMP signaling pathways function as core regulatory hubs that govern hyphal growth, morphological differentiation, and virulence regulation. In our data, the deletion of *FcLRR1* led to a loss of ability to produce macroconidia, and some abnormal conidiophore morphology was observed in the Δ*FcLRR1* mutant. Moreover, the Δ*FcLRR1* mutant presented a significantly lower germination rate than did the wild type. Conidia play essential roles in the infection process of *Fusarium* species. In *F. graminearum*, disruption of the *RAS2* gene results in delayed vegetative growth, impaired spore germination, and reduced virulence [61]. The *FgCon7* gene, which is critical for spore wall assembly and conidiogenesis, leads to decreased sporulation and germination efficiency [29]. Additionally, the transcription factor *FgSte12* is implicated in mating and hyphal development, as deletion mutants exhibit diminished conidiation and compromised pathogenicity [62]. Our data demonstrate that FcLRR1 is critical for regulating conidial development, morphological diversification, and germination efficiency in *F. circinatum*, underscoring its essential role in fungal propagation and pathogenesis.

The pathogenesis of pitch canker disease involves complex interactions between the fungal pathogen *F. circinatum* and its pine hosts. Recent multiomics studies have elucidated the virulence strategies and regulatory networks of this pathogen [63,64]. Genome-wide annotation has identified five critical pathogenicity-related genes, including Fcfga1 and Fcrho1 [35]. In subsequent studies, the RAS2 gene was shown to play a critical role in fungal growth and pathogenicity, as its knockout disrupts normal vegetative growth, reduces sporulation, and significantly reduces the virulence of *F. circinatum* [39]. In this study, we identified the *FcLRR1* gene in *F. circinatum*, the deletion of which resulted in reduced pathogenicity. Additionally, the Δ*FcLRR1* mutant presented suppressed vegetative growth and abnormal tolerance to abiotic stress. Fungal growth and development and the ability to survive under environmental stress conditions are dependent on cell wall integrity [65]. We hypothesized that the Δ*FcLRR1* mutant presented defects in cell wall integrity, leading to increased sensitivity to stress. These phenotypic defects collectively compromised the infectivity of fungal spores and hyphae toward host plants, ultimately resulting in reduced virulence. Our findings highlight the critical role of *FcLRR1* in both developmental and pathogenic processes in *F. circinatum*, offering mechanistic insights into its regulatory function in host colonization and disease progression.

In this study, transcriptome sequencing was performed on mycelial samples of the pine resin canker pathogen knockout mutant ΔFcLRR1 and its wild-type control strain A015-1. Compared with the control, the ΔFcLRR1 mutant presented 340 uniquely differentially expressed genes (DEGs, accounting for 3.90%), while 8068 DEGs were shared with the wild-type strain. A total of 555 genes were upregulated and 612 were downregulated in ΔFcLRR1 during the mycelial stage, with the number of downregulated genes exceeding that of upregulated genes. Functional enrichment analysis of DEGs was conducted via the KEGG database. KEGG analysis revealed 82 metabolic pathways, with the majority of the DEGs mapped to metabolism-related pathways (61 pathways), among which cyanoamino acid metabolism and other pathways were highly enriched. KEGG analyses revealed that the FcLRR1 protein played an important role in metabolic processes and the structural stability of the pathogen. Further investigation highlighted the significant involvement of FcLRR1 in the starch and sucrose metabolism pathways. Given the central role of this pathway in carbon source utilization and energy supply in the pathogen, we propose that FcLRR1 regulates carbon metabolism, thereby critically influencing the growth, development, stress response, and pathogenicity of the pine resin canker pathogen.

In this study, a comprehensive three-dimensional structural library of proteins for the pine resin canker pathogen was successfully constructed. Following sequence filtering (retaining sequences ≤ 800 amino acids) and OmegaFold modeling, 12,071 high-quality structural models were obtained, with most exhibiting PLDDT scores > 70. For the bait protein FcLRR1, seven key active site regions were identified via the STRING database and the PDBe website. On the basis of these regions, 100 binders were designed, and 235 candidate interacting proteins were screened through FoldSeek structural alignment (Bit score > 50). Subsequent refinement via HDock (docking score < −200) yielded 199 high-confidence candidates. Further validation with AlphaFold3 (ipTM + pTM > 0.7) identified five proteins with high interaction potential. Yeast two-hybrid assays confirmed the interaction between FcLRR1 and ALG-11, indicating that FcLRR1 is involved in carbon utilization, cell wall formation, and pathogenicity. This study provides key data and methodological references for elucidating the pathogenic mechanisms of this pathogen and developing targeted control strategies. Alpha-1,2-mannosyltransferase (Alg11) is a key enzyme in the N-glycan biosynthetic pathway within the endoplasmic reticulum (ER) of fungi and other eukaryotes. As a member of the glycosyltransferase (GT) family, this enzyme catalyzes the transfer of mannose residues from the donor substrate GDP-mannose (GDP-Man) to the growing lipid-linked oligosaccharide intermediate (Glc_3_Man_9_GlcNAc_2_-PP-dolichol). It plays an essential role in the protein N-glycosylation pathway, influencing glycoprotein folding, intracellular transport, and functional maturation. In fungi, Alg11 is critical for cell wall integrity, glycoprotein modification, immune evasion, and pathogenicity, positioning it as a significant target for studying fungal pathogenic mechanisms and for the development of novel antifungal therapeutics. For example, Biochemical Characterization and Membrane Topology of Alg2 from S. cerevisiae as a Bifunctional α1,3- and 1,6-Mannosyltransferase Involved in Lipid-linked Oligosaccharide Biosynthesis [66]. This study has several limitations. The interaction between FcLRR1 and the ORC3 protein has not been validated. ORC3 plays a critical role in cell cycle regulation, and its potential interaction with FcLRR1 will be a key focus of our future research. The present study primarily investigated the role of FcLRR1 in regulating mycelial growth, stress response, and plant infection in *F. circinatum*. Among the proteins predicted to interact with FcLRR1 by AlphaFold 3, Alg11 exhibited the highest score, leading us to prioritize its validation via yeast two-hybrid (Y2H) assay. However, given that a standalone Y2H assay cannot definitively confirm the interaction between the two proteins, additional experimental approaches will be employed in subsequent studies for further verification.

This study demonstrated that deletion of the *FcLRR1* gene impaired the growth, conidiation, and development of *F. circinatum* as well as its virulence. Given these findings and the global importance of *F. circinatum*, the results of this study will be valuable in designing management strategies that target fungal genes for the development of antifungal agents to combat pine pitch canker.

## 5. Conclusions

In this study, 13 LRR domain proteins were identified in *F. circinatum*, and *FcLRR1* was characterized by 12 tandem LRRs and an F-box domain. Phylogenetic analysis revealed its conservation in *Fusarium*, which is closest to *F. mexicanum*. qRT–PCR revealed that FcLRR1 expression peaked at 72 h postinoculation and was localized in the cytoplasm via GFP fusion. The ΔFcLRR1 mutants presented reduced growth, impaired abiotic stress tolerance, decreased conidiation (loss of macroconidia), delayed germination, and attenuated pathogenicity in young and mature seedlings, as confirmed by complementation. Thus, it can be inferred that FcLRR1 is involved in the processes of growth, development, and pathogenicity of *F. circinatum.*

## Figures and Tables

**Table 1:** Sequence and purpose of primer used in this study.

Primer Name	5-3 Direction	Purpose
GFP-F	GACGACGGCAACTACAAG	Genetic identification of the GFP transformants
GFP-R	GAACTCCAGCAGGACCAT	
hph-F	GGAGGTCAACACATCAATG	Genetic identification of the GFP transformants and deletion mutants
hph-R	CTCTATTCCTTTGCCCTCG	
FcLRR1-Up-F	CTGCCGTTCGACGATTCAGCAACAACAGATAGACAG	Upstream amplification to generate deletion vector
*FcLRR1*-Up-R	CATTGATGTGTTGACCTCCATTGTCGGTGTTGAGGGAGG	
FcLRR1-Down-F	CGAGGGCAAAGGAATAGAGATGACCGACACTTTGCTTTAG	
FcLRR1-Down-R	GGATCTTCCAGAGATTCCACCAAGGTCAAGTTCC	
FcLRR1-Out-F	AGAGATGTCAGCGAACTTG	Genetic identification of the deletion mutants
Hyg-R	GACAGACGTCGCGGTGAGTT	
Hyg-F	GTCGATGCGACGCAATCGT	
FcLRR1-Out-R	GTCTCGTTGCCATCACTC	
FcLRR1-In-F	TCCAACGACAAGAACATTAC	
FcLRR1-In-R	CGAGGTGCGACATCAATA	
L-FcLRR1-F	ACTCACTATAGGGCGAATTGGGTACTCAAATTGGTTATGGCCGCTGCTGCCGCT	Amplification of Fclrr1 to generate gene location vector
L-FcLRR1-R	CACCACCCCGGTGAACAGCTCCTCGCCCTTGCTCACGTTCATGGCGTCAGAGTGGT	
C-FcLRRr1-In-F	AGTCCTGCAACCGCCGAC	Genetic identification of the complementation transformants
C-FcLRR-In-R	GTTCATGGCGTCAGAGTGGT	
G418-F	ATGATTGAACAAGATGGAT	
G418-R	TCAGAAGAACTCGTCAAG	
Q-FcLRR1-F	GCGTGTTCATCTGAGCTACTG	Detect the expression of *Fclrr1* gene
Q-FcLRR1-R	GACAGTATGGCTGGAAGTCG	
Q-Actin-F	CCCCGTCATCATGGGATC	
Q-Actin-R	AAATCTTCTCCATGTCGTCCC	
C-FcLRR1-F	CGCTCTAGAACTAGTGGATCCGTACCTGCAGTACTTCGGGCA	Complementation of FgEC1
C-FcLRR1-R	CTTGCTCACCCTATCGAATTCGACGCAGTTCTTTCCACCAGG	

**Table 2:** Proteins with LRR Domains in *F. circinatum*.

Protein Name	Number of Amino Acids	Number of LRR Domains
FcLrr1	742	12
FcLrr2	992	8
FcLrr3	695	7
FcLrr4	1159	11
FcLrr5	2270	13
FcLrr6	378	11
FcLrr7	1831	10
FcLrr8	515	2
FcLrr9	847	1
FcLrr10	619	4
FcLrr11	976	4
FcLrr12	573	3
FcLrr13	421	8

**Table 3:** Statistics of the disease index of two-week-old slash pine seedlings infected by the wild-type strain and the knockout mutant ΔFcLRR1. * *p* < 0.05.

Strain	*DI* (5 d)	*DI* (7 d)
A015-1	70 (4.25)	83.75 (5)
Δ*FcLRR1*	12.5 (0.5) *	53.75 (3.75) *
*FcLRR-c*	67.5 (4)	81.25 (3)

**Table 4:** Sample sequencing data quality summary.

Sample Name	Clean Reads	Clean Bases	Error Rate (%)	Q20 (%)	Q30 (%)	GC Content (%)
WT-1	42,141,300	6,287,135,885	0.0128	98.21	94.81	52.84
WT-2	47,824,258	7,151,453,166	0.0126	98.28	95.01	53.16
WT-3	44,955,696	6,736,598,778	0.0128	98.2	94.79	53.19
ΔFcLRR1-1	39,991,026	5,988,702,565	0.0127	98.22	94.84	53.2
ΔFcLRR1-1	40,029,436	5,982,384,434	0.0128	98.21	94.84	52.99
ΔFcLRR1-1	40,985,642	6,138,528,765	0.0127	98.22	94.85	53.05

## Data Availability

The data underlying this article are available in the article and online Supplementary Materials.

## Supplementary Materials

The following supporting information can be downloaded at https://www.mdpi.com/article/10.3390/jof12040282/s1.
