# Supplementary figures and images for: FcLRR1 Regulates Hyphal Growth and Plant Infection in Fusarium circinatum
Source: J Fungi (Basel). 2026 Apr 16;12(4):282. doi: 10.3390/jof12040282 (PMC13117327; doi:10.3390/jof12040282)

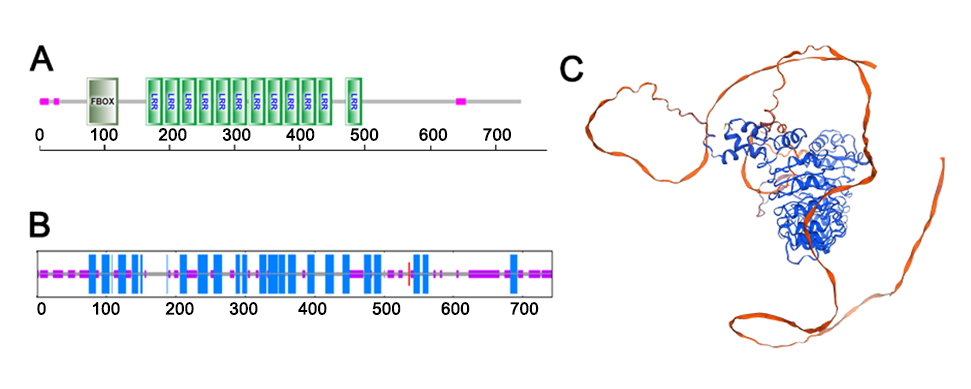

Supplement: Supplementary file 1 [file jof-12-00282-s001.zip › figure S1.tif]

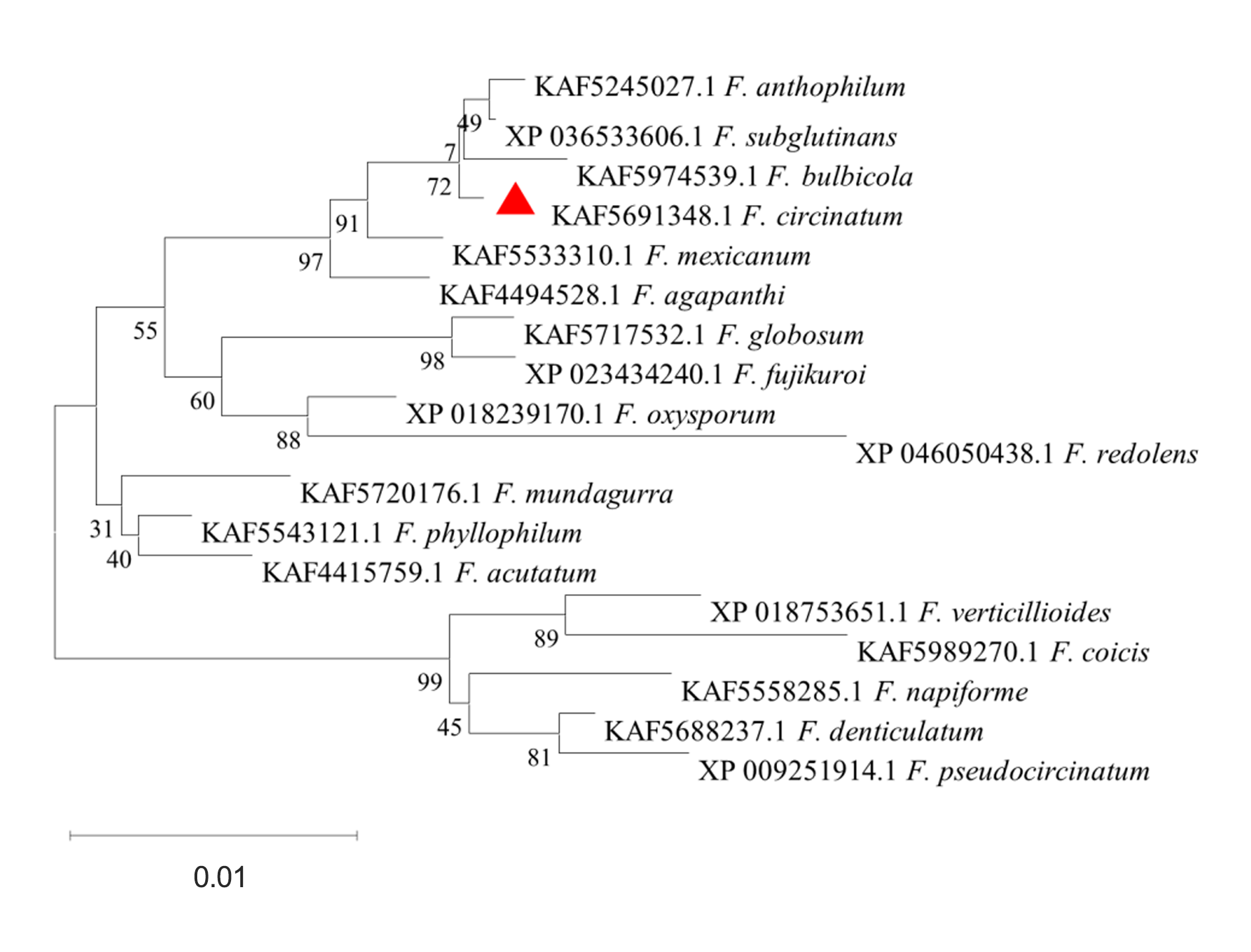

Supplement: Supplementary file 1 [file jof-12-00282-s001.zip › figure S2.tif]

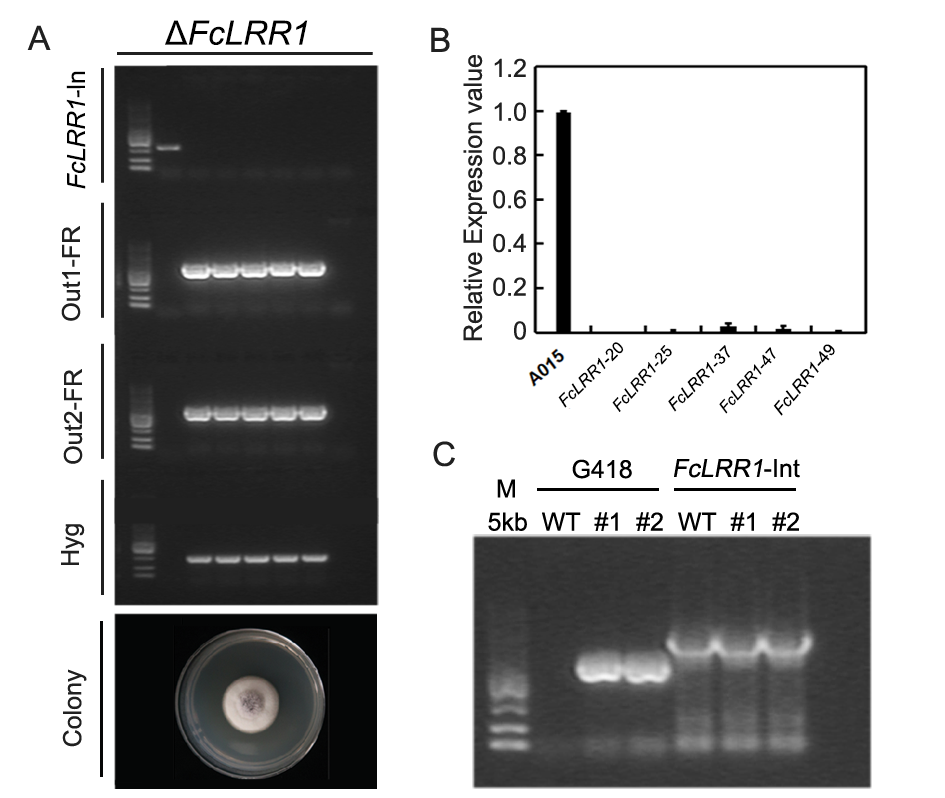

Supplement: Supplementary file 1 [file jof-12-00282-s001.zip › figure S3.tif]

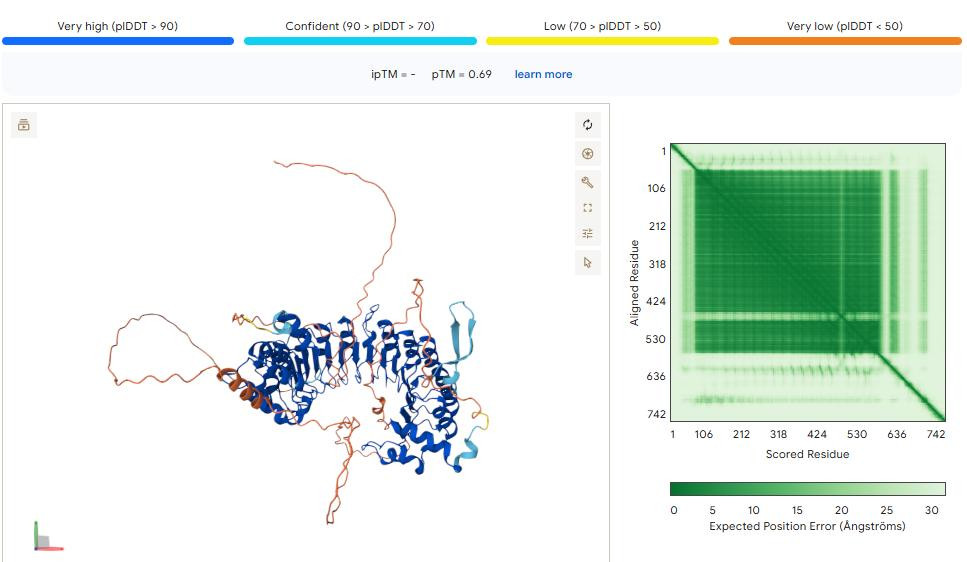

Supplement: Supplementary file 1 [file jof-12-00282-s001.zip › figure S4.jpg]

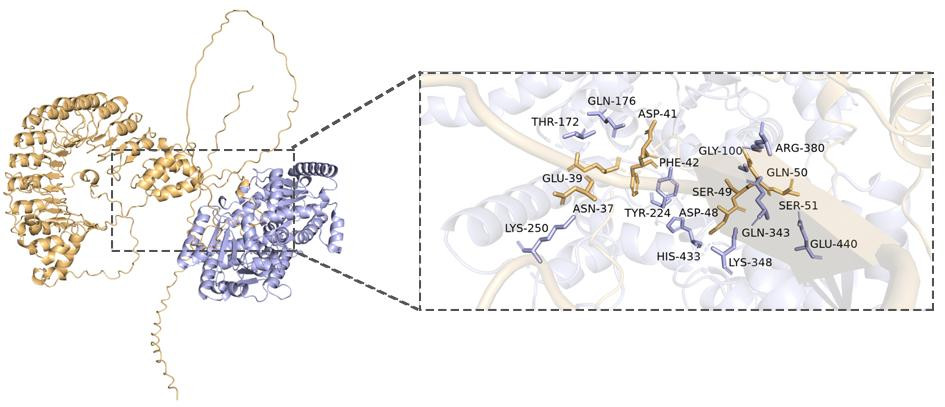

Supplement: Supplementary file 1 [file jof-12-00282-s001.zip › figure S5.jpg]
